# Multi-omics Analysis Revealed Coordinated Responses of Rumen Microbiome and Epithelium to High-Grain-Induced Subacute Rumen Acidosis in Lactating Dairy Cows

**DOI:** 10.1128/msystems.01490-21

**Published:** 2022-01-25

**Authors:** Yingyu Mu, Wangpan Qi, Tao Zhang, Jiyou Zhang, Shengyong Mao

**Affiliations:** a Ruminant Nutrition and Feed Engineering Technology Research Center, College of Animal Science and Technology, Nanjing Agricultural University, Nanjing, China; b Laboratory of Gastrointestinal Microbiology, Jiangsu Key Laboratory of Gastrointestinal Nutrition and Animal Health, National Center for International Research on Animal Gut Nutrition, College of Animal Science and Technology, Nanjing Agricultural University, Nanjing, China; University of Maine

**Keywords:** bacterial cooccurrence, cholesterol biosynthesis, high-grain diet, network analysis, rumen bacteria and epithelium, rumen metabolome

## Abstract

Subacute ruminal acidosis (SARA) is a major metabolic disease in lactating dairy cows caused by the excessive intake of high-concentrate diets. Here, we investigated the synergistic responses of rumen bacteria and epithelium to high-grain (HG)-induced SARA. Eight ruminally cannulated lactating Holstein cows were randomly assigned to 2 groups for a 3-week experiment and fed either a conventional (CON) diet or an HG diet. The results showed that the HG-feeding cows had a thickened rumen epithelial papilla with edge injury and a decreased plasma β-hydroxybutyrate concentration. The 16S rRNA gene sequencing results demonstrated that HG feeding caused changes in rumen bacterial structure and composition, which further altered rumen fermentation and metabolism. Cooccurrence network analysis revealed that the distribution of the diet-sensitive bacteria responded to the treatment (CON or HG) and that all diet-sensitive amplicon sequence variants showed low to medium degrees of cooccurrence. Metabolomics analysis indicated that the endothelial permeability-increasing factor prostaglandin E1 and the polyamine synthesis by-product 5′-methylthioadenosine were enriched under HG feeding. Transcriptome analysis suggested that cholesterol biosynthesis genes were upregulated in the rumen epithelium of HG cows. The gene expression changes, coupled with more substrate being available (total volatile fatty acids), may have caused an enrichment of intracellular cholesterol and its metabolites. All of these variations could coordinately stimulate cell proliferation, increase membrane permeability, and trigger epithelial inflammation, which eventually disrupts rumen homeostasis and negatively affects cow health.

**IMPORTANCE** Dairy cows are economically important livestock animals that supply milk for humans. The cow’s rumen is a complex and symbiotic ecosystem composed of diverse microorganisms, which has evolved to digest high-fiber diets. In modern dairy production, SARA is a common health problem due to overfeeding of high-concentrate diets for an ever-increasing milk yield. Although extensive studies have been conducted on SARA, it remains unclear how HG feeding affects rumen cross talk homeostasis. Here, we identified structural and taxonomic fluctuation for the rumen bacterial community, an enrichment of certain detrimental metabolites in rumen fluid, and a general upregulation of cholesterol biosynthesis genes in the rumen epithelium of HG-feeding cows by multi-omics analysis. Based on these results, we propose a speculation to explain cellular events of coordinated rumen bacterial and epithelial adaptation to HG diets. Our work provides new insights into the exploitation of molecular regulation strategies to treat and prevent SARA.

## INTRODUCTION

For ruminants, the diverse and symbiotic microbial community of the rumen enables its multiple metabolic functions, which are associated with animal performance and the health traits of the host (1). The rumen microbial community is evolutionarily adapted to digest and metabolize high-fiber herbivore diets. Nevertheless, in modern dairy production, cows are often fed diets containing higher proportions of concentrates or other highly fermentable carbohydrates in pursuit of ever-increasing milk production, which conversely brings about rumen microbiota dysbiosis (2, 3). The dysbiosis of the rumen microbiota could further result in rumen fermentation alterations and, subsequently, metabolic disorders, which impair cow health and, ultimately, farm profitability (4, 5).

A common metabolic disorder caused by high-concentrate feeding in dairy cows is ruminal acidosis, which can be classified as acute ruminal acidosis and subacute ruminal acidosis (SARA) according to clinical manifestations (6). Acute ruminal acidosis is characterized by prolonged exposure to a ruminal pH of ≤5.0 and a decrease in blood pH bicarbonate, which is triggered by the overproduction of ruminal d-lactic acid. In contrast, SARA has no clinical manifestations, and the ruminal pH drops to values of around 5.5 (7). Despite this, SARA appears to be more prevalent and has become a major concern within the dairy industry (8).

In recent years, the evolution of sequencing technologies has enriched the study of SARA and expanded our understanding of it. Generally, these studies reveal alterations in the structure and function of the rumen microbiota during SARA, including reduced bacterial richness and diversity, elevated levels of propionate and total volatile fatty acids (VFAs), a decreased relative abundance of fibrolytic bacteria, depressed fiber degradation, an increased amount of amylolytic bacteria, and enhanced degradation of starch (3, 9, 10). In addition, SARA challenge promotes the accumulation of various deleterious compounds such as biogenic amines and bacterial lipopolysaccharide (11, 12). Also, the structural integrity and function of the rumen epithelium are compromised during SARA, which might affect host metabolism and activate local and systemic inflammation (13, 14). Nevertheless, systematic research, from adjustments in the rumen microbiota to changes in the rumen epithelium and host metabolism, for exploring the potential coordinated responses of the rumen microbiome profile and the host to SARA challenge is rather scarce.

Therefore, the objective of our study is to investigate the microbiome-host interaction that occurs during SARA by 16S rRNA gene sequencing, liquid chromatography-mass spectrometry (LC-MS) metabolomics, and transcriptome sequencing analyses using a cow model induced by a high-grain (HG) diet. This study expands our knowledge regarding the molecular mechanisms of the cross talk between the ruminal bacterial community and the host under high-concentrate diets and will be helpful for exploiting new molecular regulation strategies to attenuate the detrimental impact of SARA on ruminants.

## RESULTS

### Rumen pH and VFA profile.

The results for rumen pH and rumen fermentation parameters were reported previously (10). In brief, HG feeding resulted in an average duration of a rumen pH of <5.8 of 9.2 h/day without affecting the ruminal lactic acid concentration (*P > *0.05), indicating the successful induction of SARA with the HG treatment. Compared to the conventional (CON) diet group, HG feeding decreased the average daily pH, the acetate concentration, the acetate ratio, and the acetate/propionate ratio (*P < *0.05), whereas it increased the concentration of total VFAs, the concentration of propionate, the propionate ratio, and the concentration of valerate (*P < *0.05). The sampling day affected the rumen pH and all of the VFA levels (*P < *0.05). The interaction between diet and day affected the rumen pH; the levels of total VFAs, acetate, propionate, and butyrate; the acetate ratio; and the propionate ratio (*P < *0.05).

### Morphological parameters of rumen epithelial papillae.

**FIG 1:**
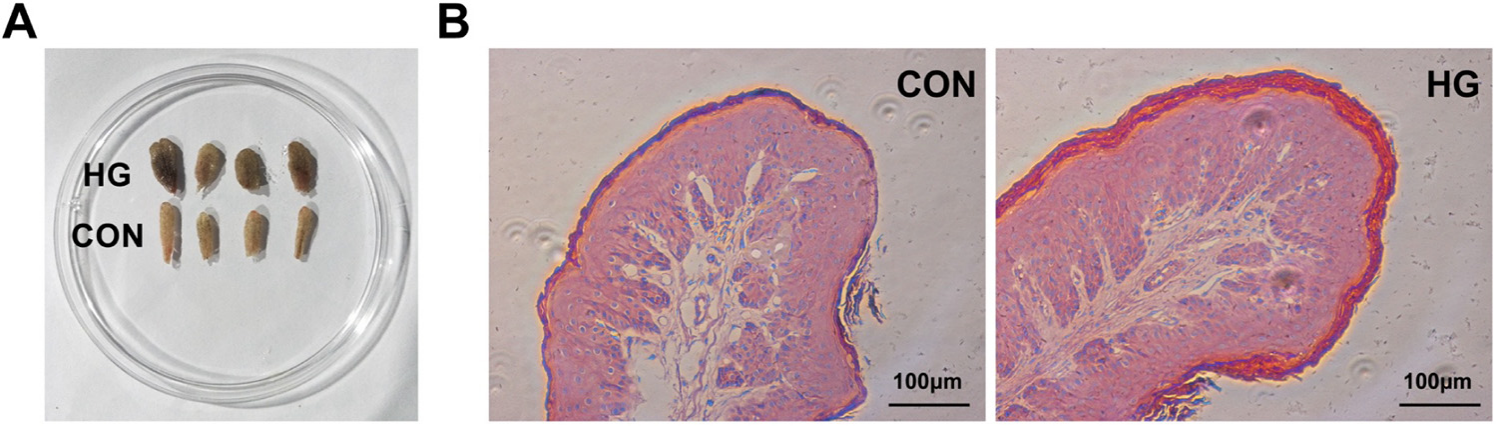
A high-grain (HG) diet affects the morphological parameters of rumen epithelial papillae. A representative visual graph (A) and light micrographs (B) of ruminal epithelial papillae in cows fed the conventional (CON) and HG diets are shown.

The rumen epithelial papillae became darkened and thickened, with the appearance of edge injury, under HG feeding (Fig. 1A). Microscopic examination of the papillae revealed increases or a trend toward increases in the thicknesses of the stratum corneum (23.09 versus 8.36 mm [*P < *0.001]), granulosum (18.66 versus 10.13 mm [*P* = 0.001]), and spinosum/basale (105.24 versus 80.60 mm [*P* = 0.054]) layers as well as the total depth of the epithelium (147.00 versus 99.09 mm [*P* = 0.011]) for the HG cows (Fig. 1B and Table 1). It also showed obvious effects of sampling day on the thicknesses of the stratum spinosum/basale and total epithelium layers (*P < *0.05). An interaction between diet and day was detected for stratum corneum thickness (*P < *0.001) (Table 1).

**TABLE 1:** Comparisons of ruminal epithelium thicknesses and blood biochemical parameters in cows fed the conventional diet and the high-grain diet^*a*^

Parameter	Value			*P* value		
	Mean for diet group		SEM			
	CON	HG		Diet	Day	Diet-day interaction
Ruminal epithelium thickness (μm)						
Total epithelium	99.09	147.00	2.59	0.011	0.023	0.066
Stratum corneum	8.36	23.09	0.49	<0.001	0.252	<0.001
Stratum granulosum	10.13	18.66	0.31	0.001	0.219	0.255
Stratum spinosum/basale	80.60	105.24	2.13	0.054	0.021	0.223

Blood biochemical parameters (concn)						
Glucose (mmol/L)	6.54	7.20	0.25	0.171	0.071	0.777
NEFAs (μmol/L)	155.70	153.13	4.41	0.793	0.682	0.779
Triglyceride (mmol/L)	1.30	1.55	0.07	0.066	0.125	0.386
Cholesterol (mmol/L)	5.38	5.33	0.18	0.893	0.870	0.659
BHBA (mmol/L)	0.44	0.39	0.02	0.009	0.086	<0.001

a CON, conventional; HG, high grain; NEFAs, nonesterified fatty acids; BHBA, β-hydroxybutyrate.

### Blood biochemical parameters.

The HG cows had a significantly lower plasma β-hydroxybutyrate (BHBA) concentration than the CON cows (0.39 versus 0.44 mmol/L [*P* = 0.009]), and a diet-week interaction was also observed for it (*P < *0.001). But the day of sampling showed no apparent effects on any of the determined plasma constituents (*P > *0.05) (Table 1).

### Effects of HG feeding on rumen bacterial structure and composition.

All 1,264,737 sequences derived from 24 samples were generated through 16S rRNA gene sequencing. A total of 1,205,373 (95%) sequences passed the quality control, with an average number of 50,224 per sample. In total, 5,512 amplicon sequence variants (ASVs) were identified across all the rarefied samples, which were affiliated with 246 genera and 19 phyla.

Compared with the CON group, HG feeding significantly reduced the number of observed ASVs (*P* = 0.008) and the Chao1 (*P* = 0.008) and Shannon (*P* = 0.008) indices but increased the Simpson index (*P* = 0.016) (see Table S1A in the supplemental material), indicating decreased bacterial richness and evenness in the HG group. The principal-coordinate analysis (PCoA) results revealed obvious discrimination in the bacterial composition between the two groups (*P* = 0.001 by permutational multivariate analysis of variance [PERMANOVA]) (Fig. S1).

10.1128/msystems.01490-21.1 FIG S1 Principal-coordinate (PCo) analysis of Bray-Curtis dissimilarities showed clustering of amplicon sequence variants between the conventional (CON) and high-grain (HG) diet groups. PERMANOVA results with 999 permutations are shown. Download FIG S1, PDF file, 0.03 MB. Copyright © 2022 Mu et al. 2022 Mu et al. https://creativecommons.org/licenses/by/4.0/ This content is distributed under the terms of the Creative Commons Attribution 4.0 International license.

10.1128/msystems.01490-21.8 TABLE S1 (A) Comparison of the α-diversity indices of the rumen bacterial community based on 16S rRNA gene sequencing. (B) Comparison of rumen bacterial compositions between the conventional (CON) and high-grain (HG) diet groups at the phylum level. (C) Properties of taxonomy assignments, amplicon sequence variant (ASV) identification numbers (ID), and log_2_ fold changes (HG versus CON) of the diet-sensitive ASVs with a mean relative abundance of >0.1%. Download Table S1, XLSX file, 0.02 MB. Copyright © 2022 Mu et al. 2022 Mu et al. https://creativecommons.org/licenses/by/4.0/ This content is distributed under the terms of the Creative Commons Attribution 4.0 International license.

We observed distinctive bacterial characteristics between the CON and HG groups at the phylum and genus levels in the predominant taxa, whose relative abundances were >1% in at least one group. At the phylum level, 19 phyla were detected in total, and the predominant phyla were comprised of the *Bacteroidota*, *Firmicutes*, *Proteobacteria*, *Spirochaetota*, *Patescibacteria*, and *Fibrobacterota*. There was a higher proportion of *Proteobacteria* (*Q *< 0.001) and lower proportions of *Bacteroidota* (*Q *= 0.027), *Spirochaetota* (*Q *= 0.027), and *Fibrobacterota* (*Q *= 0.027) in the HG group than in the CON group (Table S1B). At the genus level, a total of 246 genera were identified, and 24 abundant genera demonstrated proportions of >1%. Among them, the relative abundances of *Succinivibrionaceae* UCG-001, unclassified *Lachnospiraceae*, *Shuttleworthia*, and *Prevotella*_7 were enriched, whereas the relative abundances of the *Rikenellaceae* RC9 gut group, *Succinivibrionaceae* UCG-002, *Prevotellaceae* UCG-003, Treponema, unclassified p-251-o5, the *Christensenellaceae* R-7 group, and *Fibrobacter* were decreased in the HG group (*Q *< 0.05) (Table 2).

**TABLE 2:** Comparison of rumen bacterial compositions between the conventional and high-grain diet groups at the genus level^*a*^

Taxon	Composition (%)			*Q* value		
	Mean for diet group		SEM			
	CON	HG		Diet	Day	Diet-day interaction
*Prevotella*	28.32	21.76	1.79	0.197	0.949	0.967
*Rikenellaceae* RC9 gut group	7.10	3.48	0.51	<0.001	0.840	0.982
Unclassified F082	5.60	4.08	0.45	0.367	0.770	0.982
Unclassified *Muribaculaceae*	5.09	7.15	0.54	0.169	0.881	0.967
*Succinivibrionaceae* UCG-002	4.04	1.69	0.44	0.048	0.924	0.987
*Succiniclasticum*	3.94	4.33	0.52	0.800	0.942	0.967
*Prevotellaceae* UCG-001	3.21	2.01	0.30	0.188	0.955	0.994
*Prevotellaceae* UCG-003	2.70	0.95	0.29	0.010	0.988	0.994
Treponema	2.57	0.87	0.33	0.048	0.994	0.994
*Oscillospiraceae* UCG-005	2.33	1.88	0.24	0.291	0.958	0.987
Unclassified *Clostridia* UCG-014	2.19	3.07	0.29	0.428	0.931	0.967
*Ruminococcus*	2.05	2.69	0.27	0.400	0.830	0.999
Unclassified p-251-o5	1.90	0.40	0.20	<0.001	0.994	0.999
NK4A214 group	1.75	1.30	0.18	0.122	0.770	0.987
*Christensenellaceae* R-7 group	1.63	0.70	0.15	0.010	0.994	0.994
Unclassified *Bacteroidales* RF16 group	1.62	1.46	0.18	0.962	0.770	0.987
*Fibrobacter*	1.17	0.53	0.11	0.041	0.994	0.987
*Oscillospiraceae* UCG-002	1.12	0.63	0.11	0.154	0.770	0.967
*Prevotellaceae* UCG-004	1.00	0.66	0.09	0.255	0.958	0.999
*Succinivibrionaceae* UCG-001	0.44	11.32	1.57	<0.001	0.994	0.987
Unclassified *Lachnospiraceae*	0.28	1.48	0.23	<0.001	0.994	0.999
*Shuttleworthia*	0.07	1.03	0.15	<0.001	0.994	0.987
*Prevotella*_7	0.01	6.88	1.55	0.010	0.994	0.987
*Dialister*	0.00	1.48	0.33	0.071	0.994	0.999

a *Q* values represent the Benjamini-Hochberg-adjusted *P* values. CON, conventional; HG, high grain.

At the ASV level, ASVs with an average percentage of >0.1% made up 96% of the total ASVs. Therefore, to obtain a more convincing power, we used indicator species analysis and a likelihood ratio test to examine the ASVs sensitive to the high-grain diet effect. The results indicated that HG feeding resulted in 234 diet-sensitive ASVs (dsASVs) between the two groups (*P* < 0.05), among which 155 dsASVs had lower relative abundances in the HG cows. Moreover, 34 dsASVs showed a mean relative abundance of >0.1%, and 28 of them showed higher levels in the HG cows (Table S1C).

### Effects of HG feeding on rumen bacterial cooccurrence patterns.

**FIG 2:**
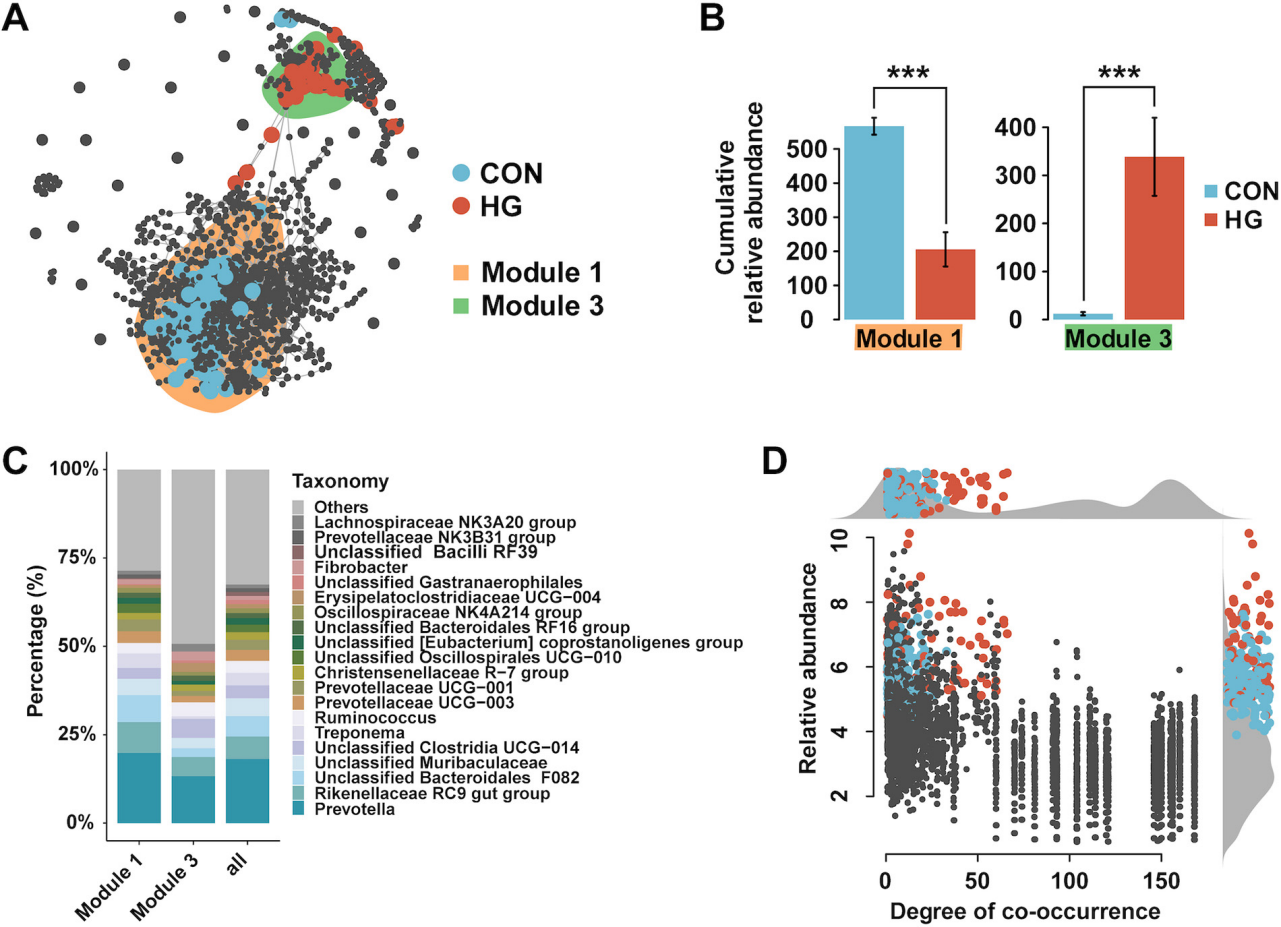
Cooccurrence patterns of diet-sensitive amplicon sequence variants (dsASVs). (A) Cooccurrence network visualizing obvious positive correlations (*r *> 0.7 and *P < *0.001) (shown with gray lines) between bacterial ASVs in the conventional (CON) and high-grain (HG) diet groups. Blue represents the dsASVs specific to the CON group, and red represents the dsASVs specific to the HG group. The ASVs that are unresponsive to either the CON or the HG group are displayed in gray. Shaded areas denote the modules that contained dsASVs in the top 10 most populated network modules. (B) Cumulative relative abundances (as counts per million [CPM] on the *y* axis [×1,000]) of all ASVs in the 2 treatment (CON or HG)-sensitive modules. ***, *P* value of <0.001 by a Wilcoxon rank sum test. (C) Taxonomic composition of the 2 treatment (CON or HG)-sensitive modules at the genus level, compared to the total taxonomic distribution of the whole data set (column “all”). (D) Degree of cooccurrence of all ASVs in the cooccurrence network. Blue represents the dsASVs specific to the CON group, red represents the dsASVs specific to the HG group, and gray represents the ASVs that are unresponsive to either the CON or the HG group. Relative abundance is expressed as log_10_-transformed CPM.

To further explore the changes in the ecological structure of bacterial communities caused by HG feeding, we next investigated the distribution patterns of the 234 dsASVs in the rumen bacterial cooccurrence network. The results showed that most of the dsASVs were primarily grouped into module 1 (M1) and module 3. Module 1 mainly involved dsASVs specific to the CON group, whereas dsASVs affiliated with the HG group were predominantly identified in module 3 (Fig. 2A). The cumulative relative abundances of all ASVs between the CON and HG groups in both module 1 and module 3 were obviously different (*P < *0.001 by a Wilcoxon rank sum test) (Fig. 2B). The phylogenetic distribution of the 2 modules was comparable to the taxonomic distribution of all ASVs, consisting of a taxonomically wide range of bacteria (Fig. 2C), which revealed that HG feeding does not target specific bacterial lineages. We determined the cooccurrence degrees for each node (number of connections to a node) and found that all dsASVs showed low to medium degrees of cooccurrence (Fig. 2D).

### Variations in rumen fluid metabolite profiles by HG feeding.

Totals of 144 and 120 metabolites were identified in the positive- and negative-ion modes, respectively. The principal-component analysis (PCA) results revealed a trend of a difference in metabolic profiles between the CON and HG groups (*P < *0.1 by PERMANOVA) (Fig. S2A and B). In the positive-ion mode of the partial least-squares discriminant analysis (PLS-DA) score plot, *R*^2^*Y* was 0.902 and *Q*^2^ was 0.232; in the negative-ion mode, *R*^2^*Y* was 0.848 and *Q*^2^ was 0.060. Although the *Q*^2^ values were low, the high *R*^2^*Y* values indicated proper goodness of fit, and the PLS-DA results showed clear separations between the 2 groups (Fig. S2C and D). The low *Q*^2^ values might be attributed to the large samples and uncontrollable individual differences. The *Q*^2^ intercept values were less than 0.05, signifying that there was no overfitting (Fig. S2E and F).

10.1128/msystems.01490-21.2 FIG S2 Multivariate analysis of rumen fluid metabolites between the conventional (CON) and high-grain (HG) diet groups. (A and B) Principal-component (PC) analysis score plots in the positive (A)- and negative (B)-ion modes. PERMANOVA results with 999 permutations are shown. (C and D) Partial least-squares discriminant analysis score plots in the positive (C)- and negative (D)-ion modes. PLS1 is the first principal component; PLS2 is the second principal component. (E and F) Permutation test plots of 200 iterations in the positive (E)- and negative (F)-ion modes. *R*^2^ and *Q*^2^ are the fitness power and the predictive power of the model, respectively. Download FIG S2, PDF file, 0.3 MB. Copyright © 2022 Mu et al. 2022 Mu et al. https://creativecommons.org/licenses/by/4.0/ This content is distributed under the terms of the Creative Commons Attribution 4.0 International license.

Based on the criteria of a variable importance in projection (VIP) score of >1 and a *P* value of <0.05, we obtained 39 differential metabolites in the positive- and negative-ion modes in total. Of these, the levels of 12 metabolites [l-ascorbic acid, prostaglandin E1 (PGE1), MG(12:0/0:0/0:0), adenine, 2-hydroxyvaleric acid, 3-hydroxysebacic acid, 5′-methylthioadenosine, deoxyribose, lysoPA(8:0/0:0), hippuric acid, palmitoleic acid, and phosphorylcholine] increased, whereas the levels of the other 27 metabolites decreased in the HG group with respect to the CON group (Table 3).

**TABLE 3:** Differential metabolites identified between the conventional and high-grain diet groups

Metabolite	VIP score^*a*^	Fold change^*b*^	*P* value
l-Ascorbic acid	1.33	9.27	0.050
PGE1	1.24	4.41	0.033
MG(12:0/0:0/0:0)	1.29	2.69	0.038
Adenine	1.84	1.98	0.005
2-Hydroxyvaleric acid	1.63	1.91	0.043
3-Hydroxysebacic acid	1.58	1.89	0.038
5′-Methylthioadenosine	1.57	1.80	0.015
Deoxyribose	1.61	1.69	0.038
LysoPA(8:0/0:0)	1.65	1.68	0.038
Hippuric acid	1.12	1.52	0.038
Palmitoleic acid	1.65	1.43	0.038
Phosphorylcholine	1.09	1.39	0.043
l-Isoleucine	1.22	0.82	0.038
3-Hydroxytetradecanedioic acid	1.66	0.82	0.033
Propenoylcarnitine	1.57	0.82	0.050
2-Octenoic acid	1.45	0.81	0.050
Sebacic acid	1.58	0.80	0.028
Suberic acid	2.06	0.77	0.015
Undecanedioic acid	2.15	0.76	0.008
Hexanoylglycine	1.49	0.74	0.050
LysoPE(0:0/16:0)	1.55	0.71	0.043
l-Acetylcarnitine	1.81	0.69	0.015
1,11-Undecanedicarboxylic acid	2.13	0.68	0.009
Leukotriene E4	1.76	0.66	0.021
3-Hexenoic acid	1.89	0.65	0.011
3′-AMP	1.79	0.65	0.018
Benzoic acid	2.03	0.61	0.018
4-Hydroxyhippuric acid	1.77	0.61	0.021
α-Tocotrienol	1.76	0.59	0.050
2′-Deoxyuridine	2.04	0.59	0.015
Spermidine	2.03	0.58	0.009
LysoPC(16:0)	1.52	0.57	0.033
γ-Tocotrienol	2.12	0.56	0.007
Choline	1.90	0.53	0.004
3-Hydroxyanthranilic acid	1.14	0.48	0.024
Homogentisic acid	1.41	0.45	0.015
LysoPE(0:0/18:0)	1.61	0.45	0.013
*N*-Acetyl-l-tyrosine	2.28	0.42	0.005
Cyclic AMP	2.73	0.41	0.000

a VIP, variable importance in projection.

b The fold change is calculated as the average level in the HG group relative to that in the CON group.

### Effects on transcriptional profiles of rumen epithelial papillae by HG feeding.

Transcriptome sequencing of 24 rumen epithelial papilla samples generated a total of 810,033,258 high-quality paired reads, with an average number of 33,751,385 reads per sample. The PCA results showed that the transcriptional profiles of the CON and HG cows were clearly separated (*P* = 0.041) (Fig. S3). We identified 596 differentially expressed genes (DEGs) between the 2 groups in total, including 344 upregulated and 252 downregulated genes with HG feeding.

10.1128/msystems.01490-21.3 FIG S3 Principal-component (PC) analysis of total genes in the rumen epithelial papillae between the conventional (CON) and high-grain (HG) diet groups. PERMANOVA results with 9,999 permutations are shown. Download FIG S3, PDF file, 0.01 MB. Copyright © 2022 Mu et al. 2022 Mu et al. https://creativecommons.org/licenses/by/4.0/ This content is distributed under the terms of the Creative Commons Attribution 4.0 International license.

**FIG 3:**
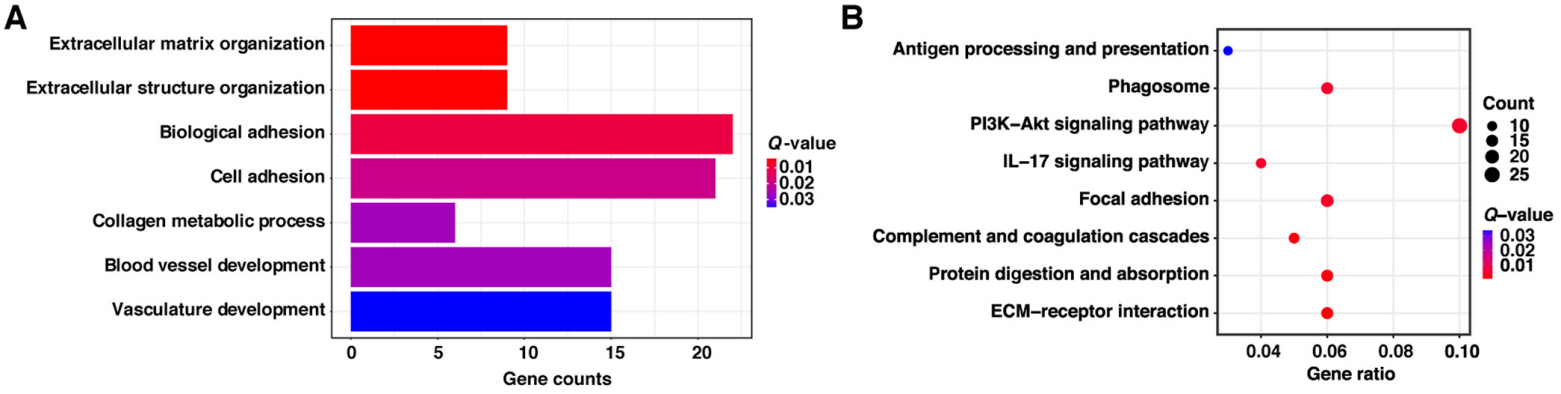
Functional enrichment analysis of differentially expressed genes (DEGs) in rumen epithelial papillae between the conventional and high-grain diet groups. (A) The significantly enriched Gene Ontology biological process terms of DEGs. (B) The significantly enriched KEGG pathways of DEGs. *Q* values represent the Benjamini-Hochberg-adjusted *P* values.

The results of the Gene Ontology (GO) enrichment of biological processes indicated that the DEGs were significantly enriched in 7 differential terms of extracellular matrix (ECM) organization, extracellular structure organization, biological adhesion, cell adhesion, collagen metabolic process, blood vessel development, and vasculature development (*Q *< 0.05) (Fig. 3A). Moreover, almost all DEGs corresponding to these terms were upregulated in the HG group (Fig. S4A). The KEGG analysis showed that the DEGs were significantly enriched in 8 pathways, including ECM-receptor interaction, protein digestion and absorption, complement and coagulation cascades, focal adhesion, the interleukin-17 (IL-17) signaling pathway, the phosphatidylinositol 3-kinase (PI3K)–Akt signaling pathway, the phagosome, and antigen processing and presentation (*Q *< 0.05) (Fig. 3B). Except for the IL-17 signaling pathway, DEGs related to the other 7 pathways were basically all upregulated in the HG group (Fig. S4B). With a visual plot of the IL-17 signaling pathway, we found that the associated DEGs were primarily involved in IL-17F-dependent signaling (Fig. S5), which exhibited a regulatory role in autoimmune disorders (15).

10.1128/msystems.01490-21.4 FIG S4 Heat-map-like functional classification of the differentially expressed genes (DEGs) in rumen epithelial papillae between the conventional (CON) and high-grain (HG) diet groups. Expression patterns of the DEGs corresponding to the significantly enriched Gene Ontology biological process terms (A) and KEGG pathways (B) are shown. The fold change is calculated as the average level in the HG group relative to that in the CON group. Download FIG S4, PDF file, 0.05 MB. Copyright © 2022 Mu et al. 2022 Mu et al. https://creativecommons.org/licenses/by/4.0/ This content is distributed under the terms of the Creative Commons Attribution 4.0 International license.

10.1128/msystems.01490-21.5 FIG S5 Visual plot of expression patterns of the differentially expressed genes related to the IL-17 signaling pathway between the conventional (CON) and high-grain (HG) diet groups. The fold change is calculated as the average level in the HG group relative to that in the CON group. Download FIG S5, PDF file, 0.3 MB. Copyright © 2022 Mu et al. 2022 Mu et al. https://creativecommons.org/licenses/by/4.0/ This content is distributed under the terms of the Creative Commons Attribution 4.0 International license.

### Weighted gene coexpression network analysis of the correlation of the host transcriptome with rumen fermentation parameters, rumen epithelial papilla morphological parameters, and blood biochemical parameters.

**FIG 4:**
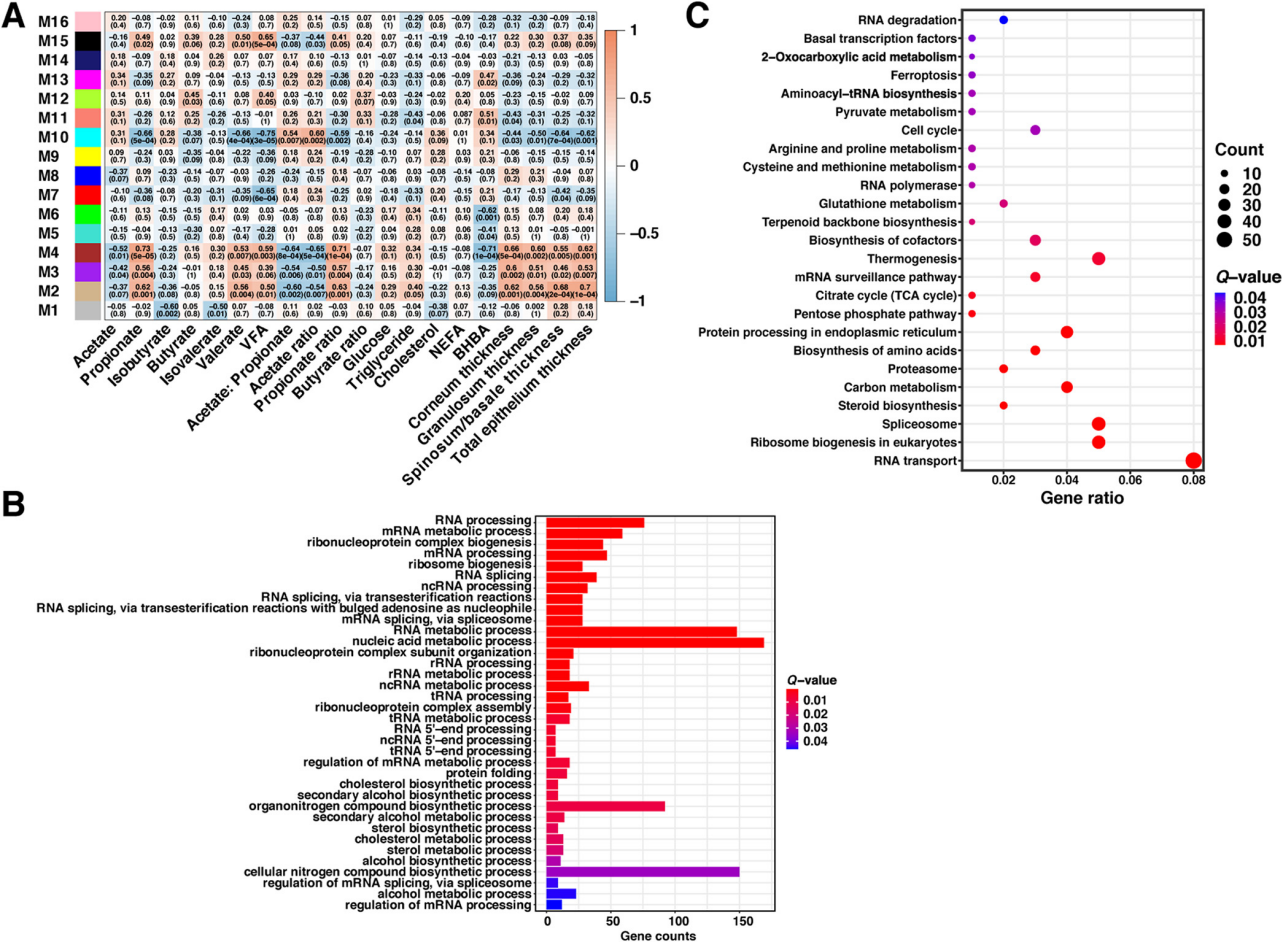
Weighted gene coexpression network analysis of the correlation of the host transcriptome with host phenotypic traits. (A) The module-trait associations. Each row corresponds to a module eigengene, with a column for each trait. Numbers in the cells indicate the correlation coefficient and *P* value; red represents a positive correlation, and blue represents a negative correlation. (B) The significantly enriched Gene Ontology biological process terms in the M4 module. ncRNA, noncoding RNA. (C) The significantly enriched KEGG pathways in the M4 module. *Q* values represent the Benjamini-Hochberg-adjusted *P* values. TCA, tricarboxylic acid.

For a comprehensive understanding of rumen epithelial transcriptome profiles under HG feeding, we further performed weighted gene coexpression network analysis (WGCNA) to identify the coexpressed gene modules that were highly correlated with host phenotypic traits. A total of 16 gene modules (M1 to M16) from all 14,078 host genes expressed in 24 samples were identified, among which M4 presented the most significant correlation (Fig. 4A). Generally, M4 was positively correlated with concentrations of propionate, valerate, and total VFAs; the propionate ratio; and ruminal epithelium thickness but was negatively correlated with the acetate concentration, the acetate/propionate ratio, the acetate ratio, and the plasma BHBA concentration (*P < *0.05) (Fig. 4A). The genes in M4 were apparently enriched in 36 GO biological process terms and 25 KEGG pathways, and almost all concerned genes were upregulated in the HG group (Fig. S6A and B). These GO terms were primarily associated with the RNA metabolic process and cholesterol biosynthetic process (Fig. 4B), while the KEGG pathways were mainly related to RNA transcription, protein synthesis, the metabolism of some amino acids and organic acids, the tricarboxylic acid cycle, RNA degradation, the proteasome, and steroid biosynthesis (Fig. 4C).

10.1128/msystems.01490-21.6 FIG S6 Heat-map-like functional classification of the genes in the M4 module. Expression patterns of the genes corresponding to the significantly enriched Gene Ontology biological process terms (A) and KEGG pathways (B) are shown. The fold change is calculated as the average level in the high-grain diet group relative to that in the conventional diet group. Download FIG S6, PDF file, 0.1 MB. Copyright © 2022 Mu et al. 2022 Mu et al. https://creativecommons.org/licenses/by/4.0/ This content is distributed under the terms of the Creative Commons Attribution 4.0 International license.

### Enhanced cholesterol biosynthesis in rumen epithelium by HG feeding.

**FIG 5:**
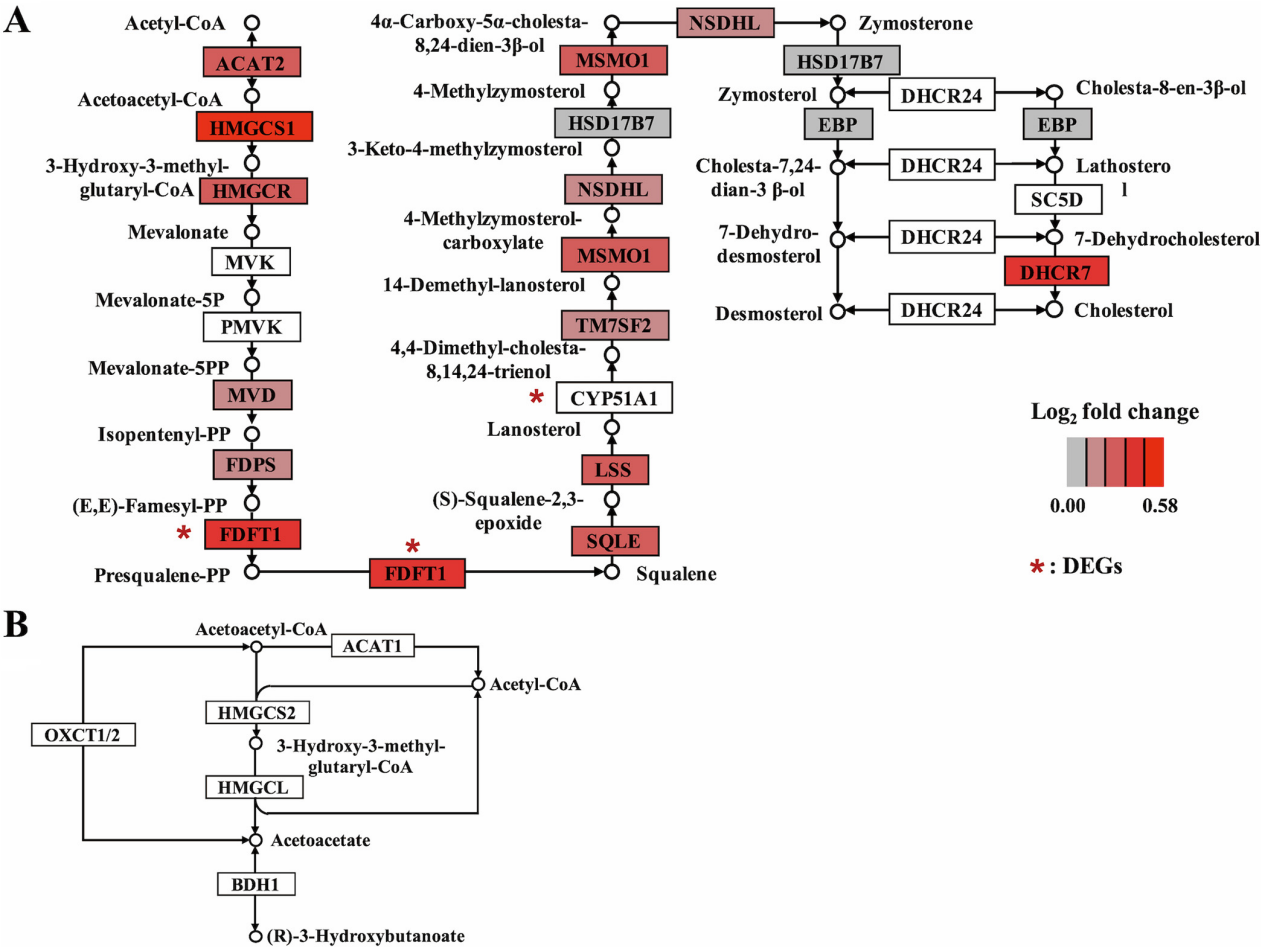
Schematic overview of ketogenesis or cholesterol biosynthesis from acetyl-CoA in rumen epithelium cells. Gene expression patterns for the cholesterol biosynthesis pathway (A) and the ketogenesis pathway (B) were compared between the conventional (CON) and high-grain (HG) diet groups. The fold change is calculated as the average level in the HG group with respect to that in the CON group. Genes with a white background indicate that the gene is not detected in the M4 module. DEGs, differentially expressed genes.

Based on the strong correlation between the host transcriptome and rumen fermentation parameters suggested by WGCNA, we next explored the changes of VFA metabolism in the rumen epithelium around the genes in M4. After being absorbed by rumen epithelial cells, the VFAs were first transformed to acetyl-CoA under the actions of a cascade of enzymes; acetyl-CoA could then further proceed to ketogenesis or cholesterol biosynthesis via different metabolic pathways. Regarding cholesterol biosynthesis, most of the genes involved in this pathway were detected in M4, and they all had higher expression levels in the HG group, including one DEG (*FDFT1*) (Fig. 5A). For the ketogenesis pathway, there were not any genes concerning ketogenesis in mitochondria identified in M4 (Fig. 5B). Moreover, none of these ketogenic genes were DEGs (*Q *> 0.05) (Table S2). In brief, HG feeding promoted cholesterol biosynthesis without affecting ketogenesis in rumen epithelial cells in lactating dairy cows.

10.1128/msystems.01490-21.9 TABLE S2 Comparison of gene expression levels of enzymes related to ketogenesis in mitochondria between the conventional (CON) and high-grain (HG) diet groups. Download Table S2, DOCX file, 0.05 MB. Copyright © 2022 Mu et al. 2022 Mu et al. https://creativecommons.org/licenses/by/4.0/ This content is distributed under the terms of the Creative Commons Attribution 4.0 International license.

## DISCUSSION

In the present study, we examined the coordinated adaptive variations between rumen bacteria and the host during SARA with combined investigations into rumen bacterial communities, rumen fluid metabolomics, and transcriptome profiling of rumen epithelial papillae. It is important to emphasize that we primarily discuss the diet effect here due to our research focus and space constraints, although the effects of sampling day and diet-week interaction were also significant.

As broadly stated previously, our results characterized a typical shift in rumen fermentation and the rumen epithelium with SARA, including higher levels of propionate and total VFAs (3, 16), a reduced level of acetate (17, 18), and a changed rumen epithelial morphology with tissue lesions (19–21). Additionally, we found an apparently decreased plasma BHBA concentration in the HG cows, which was also reported by some other researchers (22, 23).

The role of diverse microbes is the most decisive in rumen health and function. Here, we observed increased proportions of *Succinivibrionaceae* UCG-001, unclassified *Lachnospiraceae*, *Shuttleworthia*, and *Prevotella*_7 but decreased proportions of the *Rikenellaceae* RC9 gut group, *Succinivibrionaceae* UCG-002, *Prevotellaceae* UCG-003, Treponema, unclassified p-251-o5, the *Christensenellaceae* R-7 group, and *Fibrobacter* under HG feeding. Among them, the unclassified *Lachnospiraceae* and *Shuttleworthia* are members of starch-fermenting bacteria (2, 24), whereas Treponema and *Fibrobacter* are reported to be engaged in fiber degradation (25, 26). The enrichment of starch-degrading bacteria and the decreased abundance of fiber-degrading bacteria have been broadly documented in studies on SARA (9, 27); these eventually led to alterations in rumen fermentation, such as a decreased acetate level and elevated levels of propionate and total VFAs (8), which we also observed in our results. *Succinivibrionaceae* UCG-001 and *Succinivibrionaceae* UCG-002 belong to the *Succinivibrionaceae* family. Previous studies noted that *Succinivibrionaceae* UCG-001 contributed to the production of succinate (28), whereas *Succinivibrionaceae* UCG-002 was reported to be correlated with ruminal fatty acid metabolism (29). *Prevotella*_7 and *Prevotellaceae* UCG-003 belong to the *Prevotellaceae* family, within which the dominant genus *Prevotella* had been reported to represent a group of bacteria that are capable of digesting various substrates (28). As for the *Rikenellaceae* RC9 gut group, the *Rikenellaceae* family itself is a relatively new taxonomic classification, and there is a great need for more data on its metabolic function, although Su et al. proposed one acetate-producing isolate from the *Rikenellaceae* family (30). Family p-251-o5 is a “*Candidatus*” taxon with no cultured representative as of yet. The *Christensenellaceae* R-7 group is most closely related to the species Christensenella minuta (31), which can produce acetate and butyrate from glucose (32). Altogether, these results demonstrated the possibility of disturbed rumen digestion and metabolism abilities in the HG cows and explained the altered rumen fermentation parameters.

The gut microbial ecosystem is a complex and dynamic community, and it allows a collective response to various perturbations, such as environment, diet, and health, which might further affect intestinal homeostasis and metabolism (33, 34). In our study, we found that the dsASVs specific to different groups clustered into distinct modules (Fig. 2A and B), indicating treatment-specific (HG or CON) responses of the bacterial community to HG feeding. Bacterial taxa that extensively interact with other taxa in the cooccurrence network are probably core members and are believed to play a key role within the microbiome (35). Our data showed that HG feeding is primarily limited to affecting ASVs with low to medium degrees of cooccurrence despite having an obvious (*P* = 0.001) influence on community β-diversity (see Fig. S1 in the supplemental material). This finding suggests that although HG feeding causes structure fluctuation of the rumen bacterial community to some extent, it does not impact rumen bacterial taxa with keystone functions, implying the inertia and resilience of the rumen microbiota of adult ruminants to cope with certain outside perturbations (36).

Alterations in the composition and community structure of rumen bacteria lead to parallel changes in rumen fluid metabolites. One of the most interesting observations from our study was the increased levels of PGE1 and 5′-methylthioadenosine under HG feeding. PGE1 is an endothelial permeability-increasing factor (37); 5′-methylthioadenosine is a by-product of polyamine synthesis, and its accumulation can become toxic (38); and their enrichment might induce increased rumen epithelium permeability. Another interesting finding was the decreased concentrations of some beneficial compounds for the HG cows, such as α-tocotrienol, γ-tocotrienol, and choline. α-Tocotrienol and γ-tocotrienol are two different isoforms of vitamin E, which is the principal lipophilic antioxidant (39). Spermidine has pleiotropic protective effects on the organisms, such as anti-inflammatory properties, antioxidant functions, and preservation of mitochondrial function (40). Sun et al. demonstrate that rumen-protected choline can effectively alleviate oxidative stress and improve the immune function of transition dairy cows (41). We also found decreased levels of some amino acids and their derivatives (l-isoleucine, hexanoylglycine, and *N*-acetyl-l-tyrosine), suggesting that HG feeding affected protein digestion and metabolism of the cows. Besides, our data revealed a cluster of differential metabolites that are degradation products of the rumen microbiota (adenine, 5′-methylthioadenosine, 2′-deoxyuridine, and 3′-AMP) between the 2 groups; this indicated the cell lysis of certain microbiota members that could not survive the low-pH stress, which was consistent with previously noted findings on SARA (42, 43).

Accumulating evidence shows that changes in the structure and function of the intestinal microbiota affect host metabolism and immunity, especially for the luminal epithelium (44, 45). In ruminants, previous studies revealed that HG diet feeding caused an accelerated cell cycle, a promotion of cell proliferation, and a local inflammatory response of the rumen epithelium (20, 21). In this study, the results for the rumen epithelial transcriptome demonstrated that the DEGs were significantly enriched in the GO biological processes of cellular processes (extracellular matrix organization, extracellular structure organization, and cell adhesion) and system development (blood vessel development and vasculature development) as well as the KEGG pathway of signaling molecules and interaction (ECM-receptor interaction and the PI3K-Akt signaling pathway), the immune system (complement and coagulation cascades and antigen processing and presentation), and cellular processes (focal adhesion and the phagosome). Meanwhile, almost all corresponding DEGs were upregulated in the HG group. Taken together, we speculate that these enrichments mirror the improved cell cycle-regulating adaptation to maximize the absorptive surface area for increasing nutrient loads and an immune defense response of the rumen epithelium to HG feeding. This speculation corresponds to the morphological observations of rumen epithelial papillae (Fig. 1), which indicated thickening and edge injury visible to the naked eye, and microscopically enhanced ruminal epithelium thickness under HG feeding.

Further WGCNA indicated that the M4 module showed the most significant correlation with rumen fermentation parameters, ruminal epithelium thickness, and the plasma BHBA concentration. Moreover, the markedly enriched functions of the M4 module were mainly concentrated on RNA metabolic processes, protein synthesis, the metabolism of some amino acids and organic acids, the tricarboxylic acid cycle, RNA degradation, the proteasome, and steroid biosynthesis (Fig. 4B and C), with almost all corresponding genes being upregulated in the HG group. Except for steroid biosynthesis, the enrichment of these functions might also imply an accelerated cell cycle and an inflammatory event in rumen epithelial cells (46, 47), which is in agreement with the above-described enrichment results for DEGs.

In this study, the rumen VFA concentration was elevated during HG feeding; this elevation might stimulate the absorption of rumen epithelial cells (48) and promote their metabolism. VFA metabolism in the rumen epithelium predominantly includes pathways of ketogenesis and cholesterol synthesis (49). Ketogenesis in the rumen epithelium proceeds exclusively in mitochondria (14), whereas cholesterol synthesis proceeds in the cytoplasm and endoplasmic reticulum (50). Cholesterol is an essential component of mammalian cell membranes, but the excessive accumulation of cellular cholesterol and its metabolites can trigger an inflammatory response, cell proliferation, and oxidative stress and influence membrane permeability (14). In our study, we found a general upregulation of cholesterol biosynthesis genes in the rumen epithelium under HG feeding, and almost all of them were detected in M4. Collectively, the gene expression changes, in combination with more substrate being available, may have caused an enrichment of intracellular cholesterol, which could be related to well-documented symptoms of the rumen epithelium under HG diet feeding, such as an inflammation response, accelerated cell cycle, and increased membrane permeability (20, 21, 51). At the same time, it echoes the function enrichment results for the DEGs and the M4 module. However, these correlations are just our speculation and need further experimental verification in the future.

With regard to ketogenesis, we found that HG feeding did not affect the gene expression levels of ketogenic enzymes. Similarly, Steele et al. also reported the lack of differential expression of ketogenic enzyme genes using microarray analysis between cattle fed HG and those fed high-forage diets and validated this by quantitative PCR (qPCR) (14). For ruminants, acetate and ketone bodies are the main energetic substrates and can be converted to triglycerides in adipocytes and the mammary gland (52). Acetate release by the portal-drained viscera (PDV) was evidently decreased when the dietary concentrate level increased (53), which conversely would lead to a rise in the utilization of ketone bodies by peripheral tissues. Furthermore, a high metabolic cost of the rumen epithelium is expected during HG feeding owing to the stimulated VFA absorption, the accelerated outflow of rumen fluid (48), and other functional adaptations (the cell cycle and the inflammatory response). In the fed state, blood-circulating ketone bodies (acetone, acetoacetate, and BHBA) are mainly derived from synthesis by the rumen epithelium (54). The BHBA concentration is generally used as a reference for plasma ketone body levels (55). Considered altogether, it can be presumed that the decrease in the plasma BHBA concentration from our study is due to increased utilization by peripheral tissues rather than the differential gene expression levels of enzymes controlling ketogenesis in the rumen epithelium.

**FIG 6:**
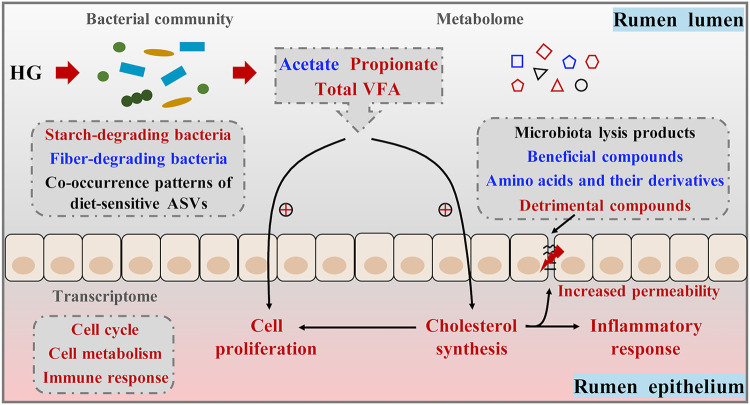
Schematic overview of the coordinated responses of rumen bacteria and epithelium to high-grain (HG) diet feeding in lactating dairy cows. Red represents upregulated levels, and blue represents downregulated levels. The plus signs represent upregulated gene expression levels. ASV, amplicon sequence variant; VFA, volatile fatty acids.

In conclusion, our study demonstrated that HG feeding caused changes in rumen bacterial profiles (structure, composition, and cooccurrence patterns of dsASVs); these changes then altered rumen fermentation, including decreased acetate concentrations and increased concentrations of propionate and total VFAs. In parallel, some detrimental metabolites were enriched, which might have increased membrane permeability. The elevated total VFA level accelerated cell proliferation of the rumen epithelium to maximize its absorptive ability and upregulated the genes for cholesterol biosynthesis. The gene expression changes, combined with more substrate being available, may have caused an accumulation of intracellular cholesterol and its metabolites, which further stimulated cell proliferation, increased membrane permeability, and triggered epithelial inflammation (Fig. 6). Additionally, due to the decreased acetate release by PDV tissues during HG feeding, there was an augmented utilization of BHBA by peripheral tissues, which resulted in a reduced plasma BHBA concentration. These findings shed new light on the coordinated microbiota-host cross talk in disturbing host homeostasis under HG feeding in lactating dairy cows.

## MATERIALS AND METHODS

All the procedures in this experiment were conducted according to animal protection law based on the *Guide for the Care and Use of Laboratory Animals* (56) and approved by the Ethics Committee of Nanjing Agricultural University.

### Animals, diets, and experimental design.

Eight ruminally cannulated (internal diameter, 10 cm) multiparous and clinically healthy lactating Holstein cows (body weight of 582 ± 50 kg; 120 ± 6 days in milk; milk yield of 18.2 ± 2.66 kg/day) were selected for the experiment. Feeding and management were described previously (10). Briefly, the animals were randomly assigned to two groups and housed in individual tie stalls during the 21-day trial period; one group was given a CON diet (40% concentrate [dry matter basis]), and the other group was given an HG diet (60% concentrate [dry matter basis]) (see Table S3 in the supplemental material). The diets were prepared as total mixed rations and formulated to meet or exceed NRC nutrient recommendations (57).

10.1128/msystems.01490-21.10 TABLE S3 Ingredients and nutritional compositions of the conventional diet (CON) and the high-grain diet (HG). Download Table S3, DOCX file, 0.05 MB. Copyright © 2022 Mu et al. 2022 Mu et al. https://creativecommons.org/licenses/by/4.0/ This content is distributed under the terms of the Creative Commons Attribution 4.0 International license.

### Rumen pH measurement, rumen content sampling, and analysis.

Rumen pH was measured at 0 h before the morning feeding and 2, 4, 6, 8, and 12 h later on days 3, 6, 7, 13, 14, 20, and 21 with a mobile pH meter (HI 99161; Hanna Instruments). The rumen content samples were collected from the ventral sac of the rumen before morning feeding and 4, 8, 12 h later on days 7, 14, and 21 via the rumen fistula. After collection, the ruminal contents were divided into 3 aliquots, 1 of which was immediately placed into liquid nitrogen for microbial DNA extraction and the other 2 of which were strained through 4 layers of sterile cheesecloth to obtain rumen fluid and frozen in liquid nitrogen or at −20°C separately for later rumen metabolome analysis and VFA quantification (10).

### Blood sampling and analysis.

Blood samples were collected via the tail vein into evacuated K_2_-EDTA (anticoagulation) tubes 6 h after the morning feeding on days 7, 14, and 21. Plasma was separated by centrifugation at 3,000 × *g* for 15 min at 4°C and stored at −80°C until analysis. The concentrations of glucose, nonesterified fatty acids (NEFAs), triglyceride, cholesterol, and BHBA in plasma were determined in duplicate using standard procedures and commercial kits (Nanjing Jiancheng Biology Research Institute, Nanjing, China).

### Rumen epithelial papilla biopsies and microscopic study.

Rumen epithelial papillae were biopsied from the ventral sac before evening feed delivery on days 7, 14, and 21, as described previously (19). First, the reticulorumen contents were partially emptied to facilitate the retraction of the ventral sac to the fistula. Next, sterile surgical scissors were used to excise approximately 200 mg of rumen papillae, and the papillae were quickly rinsed several times in ice-cold phosphate-buffered saline to remove feed particles. After washing, papillae were stored in liquid nitrogen or 4% paraformaldehyde (PFA) for subsequent transcriptome analysis or morphological observation, respectively.

Five papillae per cow per time point were prepared for light microscopy histomorphometric analysis using methods described in a previous publication (58). PFA-fixed papillae were dehydrated, paraffin embedded, sectioned, and stained with hematoxylin and eosin before being mounted for analysis. Three images were captured per papilla, including the base, middle, and tip of the rumen papilla, and a total of 15 replicates were taken per time point per cow. Image Pro Plus software (Media Cybernetics, Bethesda, MD, USA) was used to measure the thickness of each stratum (corneum, granulosum, and spinosum/basale) at a magnification of ×40 according to previously defined criteria (19).

### Microbial DNA extraction, 16S rRNA gene sequencing, and analyses.

Genomic DNAs of the rumen content samples before the morning feeding on days 7, 14, and 21 were isolated using an EZNA stool DNA kit (Omega BioTek) according to the manufacturer’s procedures, with a bead-beating step (BioSpec Products) added to break down the cell walls of the microbes. The concentration and quality of the extracted DNA were examined using a NanoDrop 1000 spectrophotometer (Nyxor Biotech, Paris, France) and 1.0% agarose gel electrophoresis, respectively. The V3-V4 region of the 16S rRNA gene was amplified using the 341 forward primer (5′-CCTAYGGGRBGCASCAG-3′) and the 806 reverse primer (5′-GGACTACNNGGGTATCTAAT-3′) (59). A total of 27 cycles of PCR amplification were conducted. The PCR products were purified with the GeneJET gel extraction kit (Thermo Scientific). Amplicon libraries were constructed using a New England BioLabs (NEB) Next Ultra DNA library prep kit for Illumina and sequenced (paired end, 2 by 250 bp) on the Illumina MiSeq platform (Illumina Inc.).

The raw sequences were demultiplexed using an in-house Perl script based on their unique barcodes and processed to filter low-quality reads with the following criteria: the 250-bp reads were truncated at any site receiving an average quality score of <20 over a 10-bp sliding window, discarding the truncated reads that were shorter than 50 bp. Paired-end reads were then merged using FLASH v1.2.7 (60), with a minimum 10-bp overlap. The sequences were further screened to remove chimeras using Vsearch software (v2.18.0) (61), followed by dereplication and ASV feature table construction using the DADA2 (62) plug-in implemented in QIIME 2 v2021.08 (63). Taxonomy was assigned to ASVs using the naive Bayes classifier (64) trained against the SILVA v138 database (65) trimmed to match the V3-V4 region sequenced. To control for intersample depth variability, all samples were rarefied to 23,278 reads, the size of the smallest sample. Rarefaction curves were generated to ensure adequate sequencing depth (Fig. S7). The rarefied ASV count table was used for analyses of α-diversity, β-diversity, and taxonomic classification. α- and β-diversity metrics were calculated using the q2-diversity plug-in in QIIME 2. β-Diversity was assessed using Bray-Curtis dissimilarity and visualized by a PCoA plot. The statistical significance of the PCoA was determined using the adonis function in the R package vegan (v2.5-7) with 999 permutations.

10.1128/msystems.01490-21.7 FIG S7 Rarefaction curves based on amplicon sequence variants (ASVs). Each curve represents one individual sample. CON, conventional diet; HG, high-grain diet. Download FIG S7, PDF file, 0.04 MB. Copyright © 2022 Mu et al. 2022 Mu et al. https://creativecommons.org/licenses/by/4.0/ This content is distributed under the terms of the Creative Commons Attribution 4.0 International license.

### dsASV identification and bacterial cooccurrence network construction.

We employed indicator species analysis with the R package indicspecies (v1.7.9) and a likelihood ratio test with the R package edgeR (v3.32.1), which were more sensitive statistical tests than standard nonparametric tests (66), to identify the ASVs responsible for the high-grain diet effect. The ASV sequence counts were normalized using the “trimmed means of M” method and were expressed as relative abundance counts per million in advance (67). Only ASVs that were confirmed by both the indicator species analysis and the likelihood ratio test simultaneously were considered to have a significant difference between groups, and we defined those ASVs as dsASVs. Statistical significances were declared at *P* values of <0.05.

The bacterial cooccurrence network was constructed using the R package igraph (v1.2.6) with the Fruchterman-Reingold layout with 9,999 permutations. Relationships between ASVs were tested with the Spearman rank correlation utilizing the normalized counts per million, and positive correlations (*r* > 0.7 and *P < *0.001) were visualized. Network module identification was conducted using a fast-greedy modularity optimization procedure (68).

### Rumen metabolome analysis.

Twenty-four rumen fluid samples obtained before the morning feeding on days 7, 14, and 21 were slowly thawed at 4°C, and from each sample, a 100-μL aliquot was taken to mix with 300 μL of methanol and 10 μL of the internal standard (2.8 mg/mL l-2-chlorophenylalanine). The mixture was vortexed for 30 s and maintained at −20°C for 60 min, followed by centrifugation at 13,800 × *g* for 10 min at 4°C. The resulting supernatant was collected for analysis.

The LC-MS analysis was performed using an Ultimate 3000LC-Q-Exactive instrument (Thermo, CA, USA) incorporating a Hyper gold C_18_ column (100 mm by 2.1 mm, 1.9 μm; Thermo). The column temperature was maintained at 40°C. The mobile phase consisted of mobile phase A (water plus 5% [vol/vol] acetonitrile and 0.1% [vol/vol] formic acid) and mobile phase B (acetonitrile plus 0.1% [vol/vol] formic acid) at a flow rate of 0.3 mL/min. The elution procedure was as follows: 5% mobile phase B from 0 to 1 min, 5% to 95% mobile phase B from 1 to 11 min, and 95% to 5% mobile phase B from 11 to 19.5 min. The injection volume was 10 μL, and the autosampler was maintained at 4°C. The mass spectrometric settings for positive/negative-ion modes were as follows: a heater temperature of 300°C, a sheath gas flow rate of 45 arb, an auxiliary gas flow rate of 15 arb, a sweep gas flow rate of 1 arb, a spray voltage of 3.0 kV/3.2 kV, a capillary temperature of 350°C, and an S-lens radio frequency (RF) level of 30%/60%, respectively.

The raw data were analyzed with feature extraction and preprocessing by Compound Discoverer 2.0 software (Thermo Scientific). Ion peak data that were present in <50% of the samples were removed. The main parameters were set as follows: an intensity threshold of 300,000, an *m/z* range of 70 to 1,050, an *m/z* width of 5 ppm, a frame time width of 0.2 min, and retention time start and end values of 0.01 and 19.5 min, respectively. Next, they were normalized according to the interior label and postedited in Excel 2010 software. The online Human Metabolome Database (https://hmdb.ca) and KEGG database (https://www.genome.jp/kegg/) were used to identify metabolites by aligning the molecular mass data. The metabolites were reported only when the difference between the theoretical mass and the observed mass was <20 ppm and further validated by isotopic distribution measurement. PCA, PLS-DA, and loading plots were carried out using SIMCA-P software (version 13.0; Umetrics, Umea, Sweden). The PLS-DA models were validated based on the variation interpretation (*R*^2^*Y*) and predictability (*Q*^2^) of the model in cross-validation and permutation tests with 200 iterations. Differential metabolites were identified according to the VIP obtained from the PLS-DA model and statistical analysis (VIP score of >1 and *P* value of <0.05). When the metabolites were identified in both positive- and negative-ion modes, the data in the mode with the lower *P* value were retained.

### Epithelial RNA extraction and sequencing.

Total RNA of rumen epithelial papillae from 24 samples obtained before evening feed delivery on days 7, 14, and 21 was extracted using TRIzol (TaKaRa Bio, Otsu, Japan). The RNA concentration was measured using a NanoDrop 1000 spectrophotometer (Thermo Fisher Scientific, Waltham, MA, USA). RNA quality was evaluated with an Agilent 2100 bioanalyzer (Agilent Technologies, San Diego, CA, USA), and the RNA integrity number was ≥8.5 for all samples. The sequencing libraries were prepared with 1 μg of high-quality total RNA using an Illumina TruSeq RNA sample preparation kit (Illumina, San Diego, CA, USA) to enrich poly(A)-tailed host mRNA with oligo(dT) beads, and sample-specific barcodes were added. After quantification by a TBS 380 fluorometer (Turner Biosystems, Sunnyvale, CA, USA), the paired-end libraries (2 by 150 bp) were sequenced on the Illumina NovaSeq 6000 platform.

### Transcriptome analyses.

The raw sequences were filtered to remove adaptor contaminations and low-quality reads using Trimmomatic v0.36 (69). Next, clean reads were aligned to the Bos taurus reference genome ARS-UCD1.2 using HISAT2 with default parameters (70), and HTSeq v0.11.1 was used to count the reads mapped to each gene (71). Differential expression analyses were conducted with DESeq2 v1.30.1 (72). DEGs were screened with a cutoff condition of an absolute fold change of >1.2 and a Benjamini-Hochberg-adjusted *P* value (*Q*) of <0.05.

The R package WGCNA v1.70-3 was employed to identify trait-related gene coexpression modules (73). Gene expression data were prefiltered by keeping genes with at least one read across all 24 samples and then normalized by transcripts per kilobase per million (TPM) and log_2_ transformed [log_2_(TPM + 1)]. We use the automatic one-step function blockwiseModules to perform network construction and module detection with the following parameters: power of 14, maxBlockSize of 15,000, minModuleSize of 30, signed network type, mergeCutHeight of 0.25, and default parameters for other settings. Because of the normal distribution of the data (Shapiro-Wilk test), module-trait relationships were calculated by Pearson correlation analysis.

Principal-component analysis was performed using the prcomp function of R (version 4.0.5). GO enrichment and KEGG analyses were implemented using the R package clusterProfiler v3.18.1 (74), and biological categories were considered significant at a *Q* value of <0.05.

### Statistical analyses.

The linear mixed-effects model (MIXED) procedure of IBM SPSS statistics V25.0 (IBM Corp., Armonk, NY, USA) was performed to assess the differences in rumen pH, VFAs, blood biochemical parameters, and ruminal epithelium thickness. The treatment (CON or HG), day, and their interaction were treated as fixed factors. The cow was considered a random effect, and the hour or replicates of rumen papillae were considered a repeated measure. Effects were deemed significant when the *P* value was <0.05.

The data on α-diversity indices, relative abundances of bacterial communities, and the rumen fluid metabolome were analyzed using the nonparametric Scheirer-Ray-Hare extension of the Kruskal-Wallis test (75), which is a nonparametric analog of analysis of variance (ANOVA) based on ranked variates with two independent factors (group and day) plus their interactions. For rumen bacterial abundance, differences were regarded as being significant at a *Q* value of <0.05.

### Data availability.

Raw reads from rumen content 16S rRNA gene sequencing were deposited in the NCBI SRA database under accession number PRJNA761857. Raw reads from rumen epithelium transcriptome sequencing were deposited in the NCBI SRA database under accession number PRJNA762113. Metabolomics data were deposited in the CNSA (https://db.cngb.org/cnsa/) of the CNGBdb under accession number CNP0002475.
