# Supplementary figures and images for: Multi-omics Analysis Revealed Coordinated Responses of Rumen Microbiome and Epithelium to High-Grain-Induced Subacute Rumen Acidosis in Lactating Dairy Cows
Source: mSystems. 2022 Jan 25;7(1):e01490-21. doi: 10.1128/msystems.01490-21 (PMC8788321; doi:10.1128/msystems.01490-21)

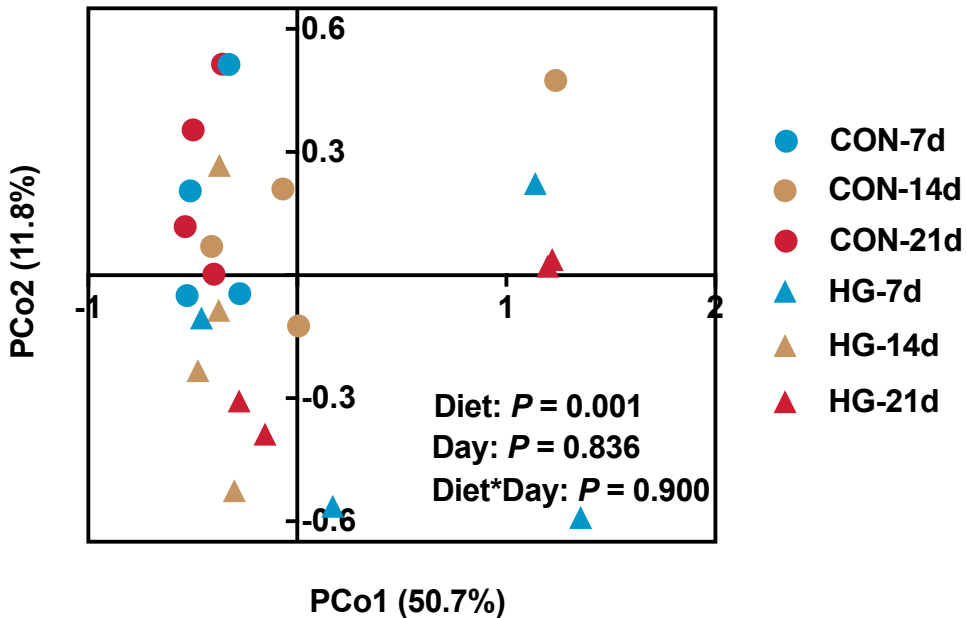

Supplement: FIG S1 [file msystems.01490-21-sf001.pdf]

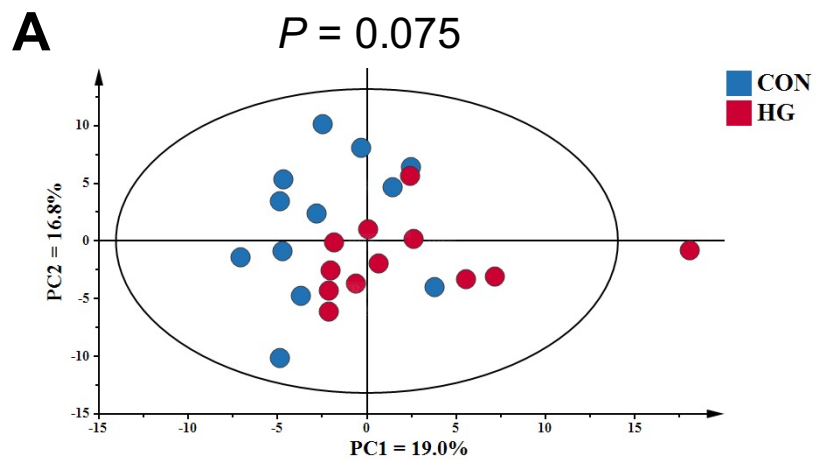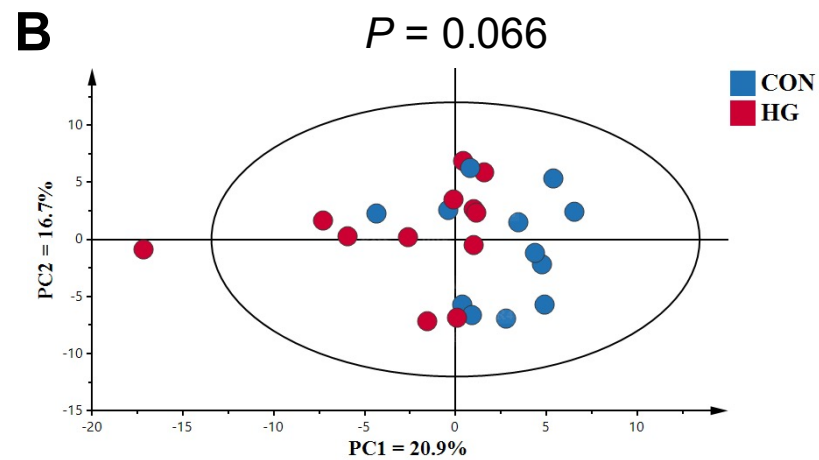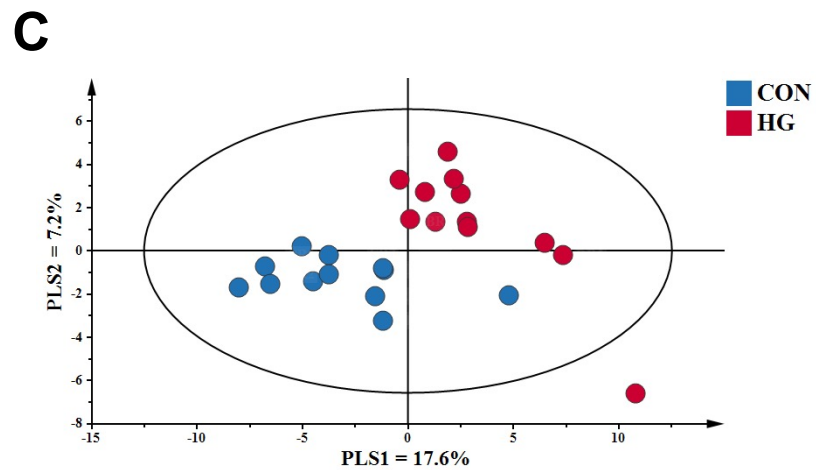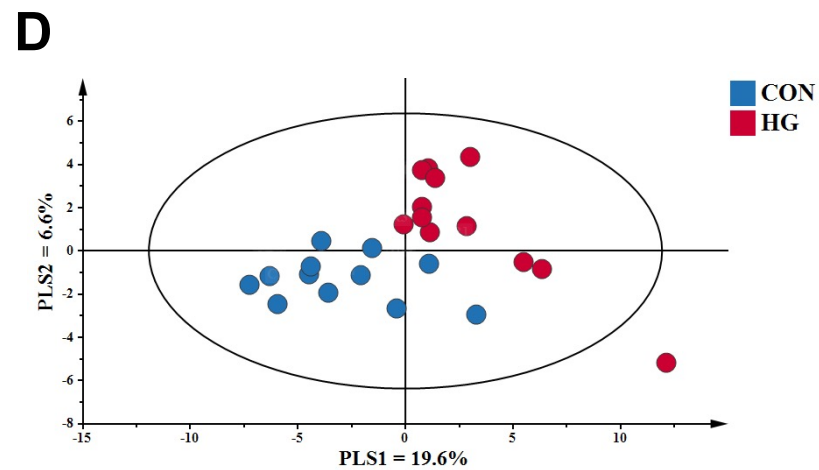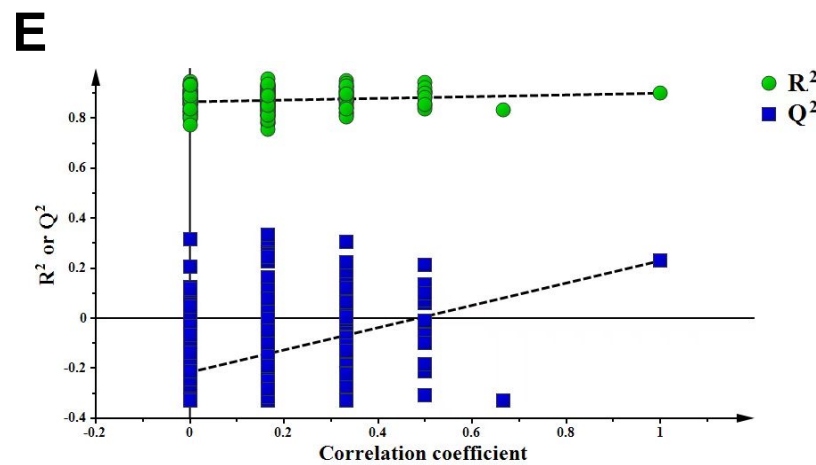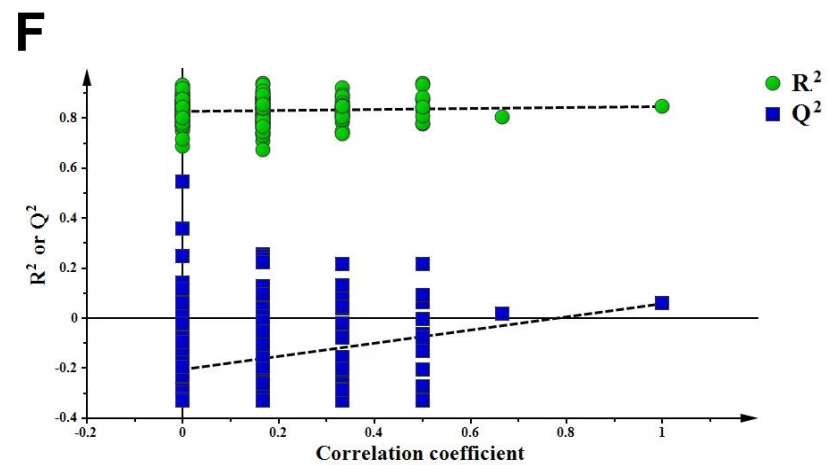

Supplement: FIG S2 [file msystems.01490-21-sf002.pdf]

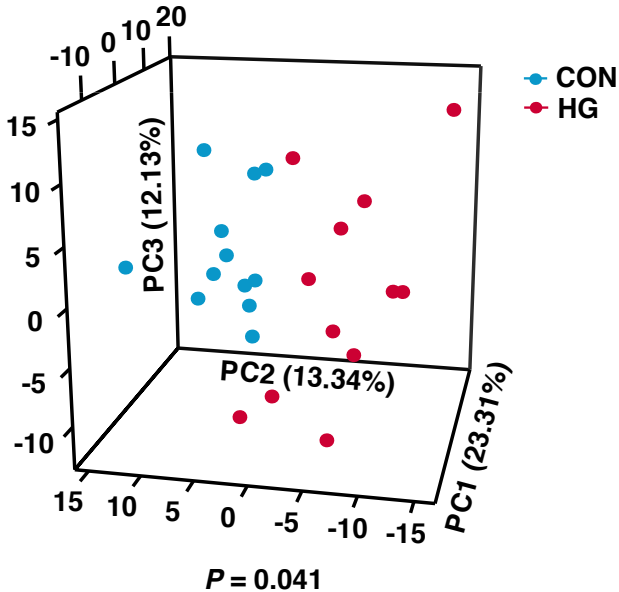

Supplement: FIG S3 [file msystems.01490-21-sf003.pdf]

# B

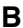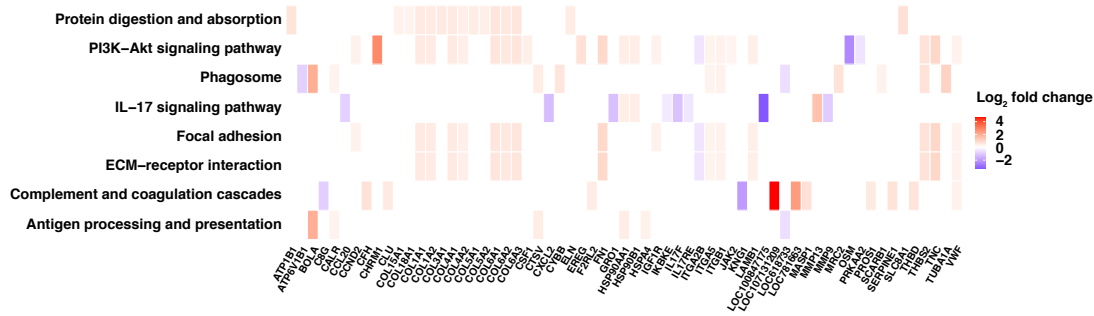

Supplement: FIG S4 [file msystems.01490-21-sf004.pdf]

**Log<sub>2</sub> fold change**

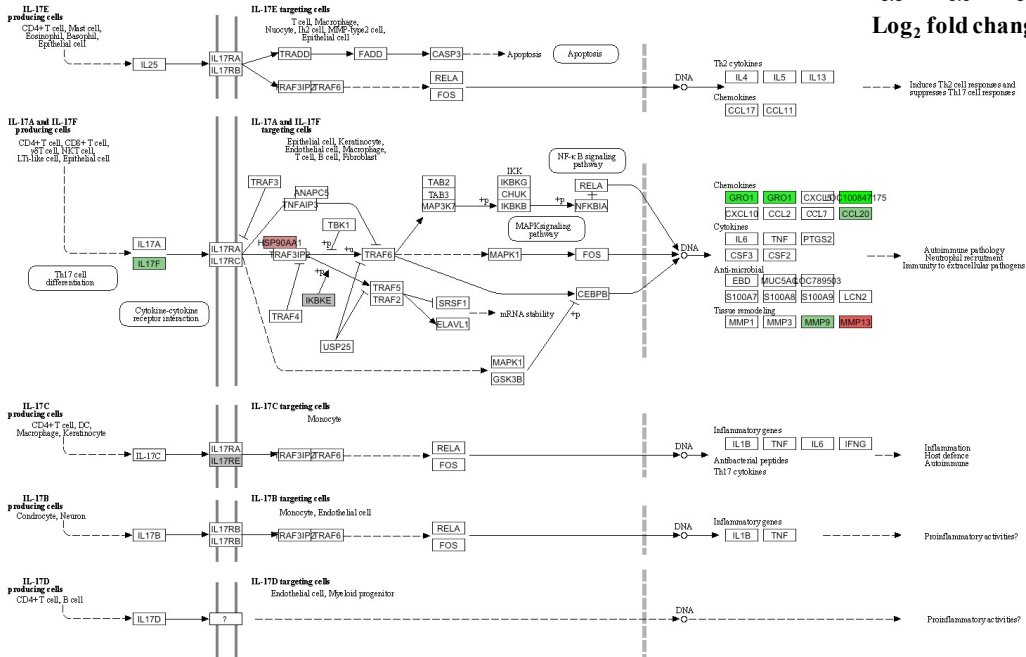

Supplement: FIG S5 [file msystems.01490-21-sf005.pdf]

**A**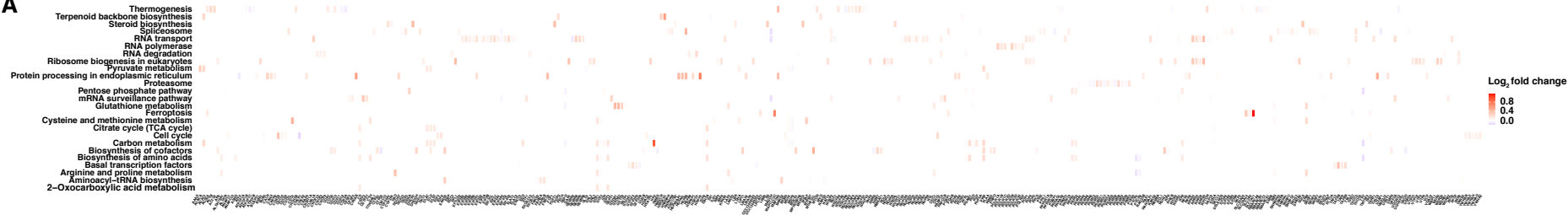**B**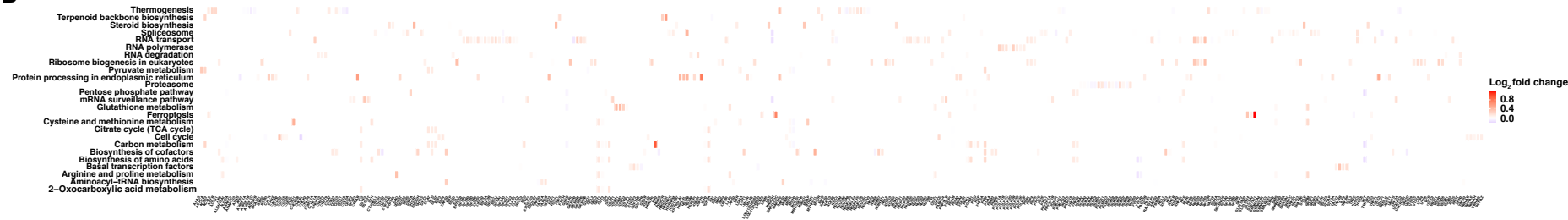

Supplement: FIG S6 [file msystems.01490-21-sf006.pdf]

Number of ASVs

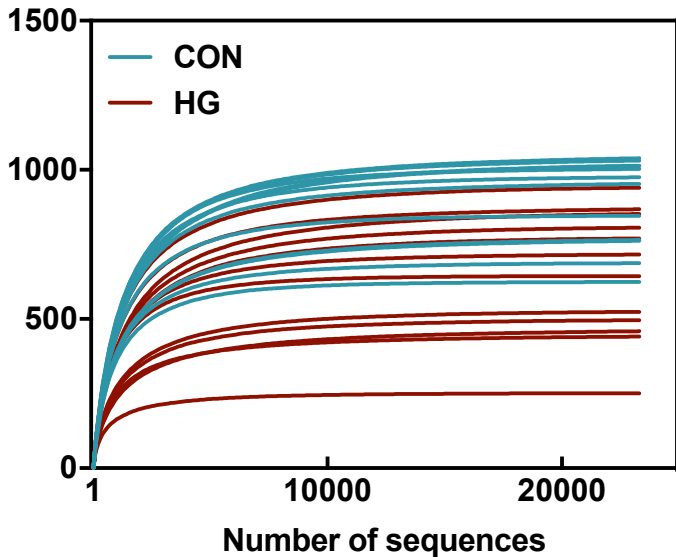

Supplement: FIG S7 [file msystems.01490-21-sf007.pdf]
