# Supplementary material for: Multi-omics Analysis Revealed Coordinated Responses of Rumen Microbiome and Epithelium to High-Grain-Induced Subacute Rumen Acidosis in Lactating Dairy Cows
Source: mSystems. 2022 Jan 25;7(1):e01490-21. doi: 10.1128/msystems.01490-21 (PMC8788321; doi:10.1128/msystems.01490-21)
Supplement: TABLE S2 [file msystems.01490-21-st002.docx]

Table S2. Comparison in gene expression levels of enzymes related to ketogenesis in mitochondria between the conventional (CON) and high-grain (HG) diet groups

| Genename | *P-*value | *Q-*value | Log_2_ fold change |
| --- | --- | --- | --- |
| *ACAT1* | 0.129 | 0.435 | -0.13 |
| *HMGCS2* | 0.811 | 0.931 | -0.02 |
| *HMGCL* | 0.880 | 0.958 | -0.01 |
| *BDH1* | 0.031 | 0.214 | -0.20 |
| *OXCT1* | 0.077 | 0.343 | 0.25 |
| *OXCT2* | - | - | - |

*^a^* Fold change is calculated as the average level in the HG group with respect to that in the CON group.

*^b^* The “-” represents it is not detected in the present study. *Q*-value represents the Benjamini-Hochberg adjusted *P*-value.
