# Supplementary material for: Multi-omics Analysis Revealed Coordinated Responses of Rumen Microbiome and Epithelium to High-Grain-Induced Subacute Rumen Acidosis in Lactating Dairy Cows
Source: mSystems. 2022 Jan 25;7(1):e01490-21. doi: 10.1128/msystems.01490-21 (PMC8788321; doi:10.1128/msystems.01490-21)
Supplement: TABLE S3 [file msystems.01490-21-st003.docx]

Table S3. Ingredients and nutritional composition of the conventional diet (CON) and the high-grain diet (HG)

| Item | CON | HG |
| --- | --- | --- |
| Ingredients, % of DM |  |  |
| Corn grain | 19.40 | 24.92 |
| Soybean | 13.50 | 13.48 |
| Barley | — | 12.00 |
| DDGS | 3.80 | 5.91 |
| CaCO3 | 0.80 | 1.48 |
| Ca(HCO₃)₂ | 1.10 | 0.92 |
| NaCl | 0.40 | 0.37 |
| Premix | 1.00 | 0.92 |
| Corn silage | 12.00 | 6.00 |
| American alfalfa hay | 24.00 | 17.00 |
| Australian oaten hay | 24.00 | 17.00 |
| Nutrients composition |  |  |
| DM, % | 46.77 | 48.03 |
| CP, % of DM | 16.16 | 16.12 |
| Crude fat, % of DM | 3.05 | 3.05 |
| NDF, % of DM | 36.14 | 29.92 |
| NFC, % of DM | 38.68 | 46.04 |
| Starch, % of DM | 17.96 | 27.82 |
| Ash, % of DM | 5.97 | 4.87 |
| Ca, % of DM | 1.14 | 1.18 |
| P, % of DM | 0.52 | 0.51 |
| NE_L_ (Mcal/kg of DM) | 1.57 | 1.64 |
| NFC/NDF | 0.93 | 1.54 |

*^a^* DDGS = Dried distillers grains with solubles; DM = dry matter; CP = crude protein; NDF = neutral detergent fiber; NFC = nonfiber carbohydrates; NE_L_ = net energy for lactation.

*^b^* Premix contained the following ingredients per kilogram of diet: vitamin A, 22.5 KIU/kg; vitamin D3, 5.0 KIU/kg; vitamin E, 37.5 IU/kg; vitamin K3, 5.0 mg/kg; Mn, 63.5 mg/kg; Zn, 111.9 mg/kg; Cu, 25.6 mg/kg; and Fe, 159.3 mg/kg.

*^c^* NFC = 100 – (% NDF + % CP + % ether extract + % ash).

*^d^* NE_L_ was calculated based on Ministry of P. R. China recommendations (MOA, 2004).

**REFERENCES**

MOA (Ministry of Agriculture of PR China). 2004. Feeding Standard of Dairy Cattle (NY/T 34–2004). MOA, Beijing, China.
